# CRISPR/Cas9-Mediated Genome Editing in Comfrey (*Symphytum officinale*) Hairy Roots Results in the Complete Eradication of Pyrrolizidine Alkaloids

**DOI:** 10.3390/molecules26061498

**Published:** 2021-03-10

**Authors:** Mahmoud M. Zakaria, Brigitte Schemmerling, Dietrich Ober

**Affiliations:** 1 Botanisches Institut und Botanischer Garten, Christian-Albrechts-Universität zu Kiel, D-24098 Kiel, Germany; mmohamed@bot.uni-kiel.de (M.M.Z.); bschemmerling@bot.uni-kiel.de (B.S.); 2 Department of Pharmacognosy, Faculty of Pharmacy, Zagazig University, 44519 Zagazig, Egypt

**Keywords:** alkaloid biosynthesis, GC-MS, HPLC, polyamine analytics, genome editing, CRISPR/Cas9

## Abstract

Comfrey (*Symphytum officinale*) is a medicinal plant with anti-inflammatory, analgesic, and proliferative properties. However, its pharmaceutical application is hampered by the co-occurrence of toxic pyrrolizidine alkaloids (PAs) in its tissues. Using a CRISPR/Cas9-based approach, we introduced detrimental mutations into the *hss* gene encoding homospermidine synthase (HSS), the first pathway-specific enzyme of PA biosynthesis. The resulting hairy root (HR) lines were analyzed for the type of gene-editing effect that they exhibited and for their homospermidine and PA content. Inactivation of only one of the two *hss* alleles resulted in HRs with significantly reduced levels of homospermidine and PAs, whereas no alkaloids were detectable in HRs with two inactivated *hss* alleles. PAs were detectable once again after the HSS-deficient HRs were fed homospermidine confirming that the inability of these roots to produce PAs was only attributable to the inactivated HSS and not to any unidentified off-target effect of the CRISPR/Cas9 approach. Further analyses showed that PA-free HRs possessed, at least in traces, detectable amounts of homospermidine, and that the PA patterns of manipulated HRs were different from those of control lines. These observations are discussed with regard to the potential use of such a CRISPR/Cas9-mediated approach for the economical exploitation of in vitro systems in a medicinal plant and for further studies of PA biosynthesis in non-model plants.

## 1. Introduction

Several traditional medicinal plants contain, in addition to metabolites with various valuable nutritional and therapeutic properties, a structurally diverse group of compounds called the pyrrolizidine alkaloids (PAs) [1,2], many of which are regarded as being toxic and having the potential to cause human poisoning [3]. Therefore, despite the beneficial characteristics of their valuable metabolites, the medicinal use of these herbs has been limited by several organizations such as BfArM, IPCS, FDA, and AHPA because of the reported toxicity of PAs [4,5,6,7]. Numerous medicinal herbs are restricted for oral consumption as their PA levels exceed the maximum tolerance levels defined for pharmaceuticals. Accordingly, the effect of such a restrictive policy is that, whenever a natural compound is shown to have some level of toxicity, as is the case for PAs, all medicinal herbs containing that compound are excluded from use. This limits our ability to make use of a natural health-care approach. At present, sophisticated extraction or purification procedures can be applied to reduce the level of toxic PAs in preparations of PA-containing plants, but these methods are laborious and expensive [8,9]. New strategies might help to optimize the clinical and economic benefits of herbal medicine by minimizing its toxic and hazardous effects, e.g., by reducing or ultimately shutting down PA-production within in vitro systems.

One such medicinal plant that contains PAs is comfrey (*Symphytum officinale* L., Boraginaceae), which is regarded as being an effective phytopharmaceutical for humans because of its anti-inflammatory, analgesic, and proliferative properties [10]. Over the centuries, preparations from comfrey root and other parts of the plant have been utilized for a wide range of medicinal indications—in particular for muscle injuries, sprains, and osteoarthritis—with effects that seem to be superior to those of conventional medicines and that expand the considerable economic importance of this plant species [11]. Allantoin and other phenolic compounds, mainly rosmarinic acid, are considered to be responsible for its main biological activities [12]. However, because of the high content of PAs present in the whole plant, most comfrey preparations are licensed only for external applications for safety reasons [13]. One single oral administration of PA-containing preparations is sufficient to result in the metabolic activation of the PAs in the liver by hepatic microsomal cytochrome P450-dependent monooxygenases and the subsequent formation of highly active pyrrolic intermediates that induce hepatotoxic or genotoxic effects [14,15].

**Figure 1:**
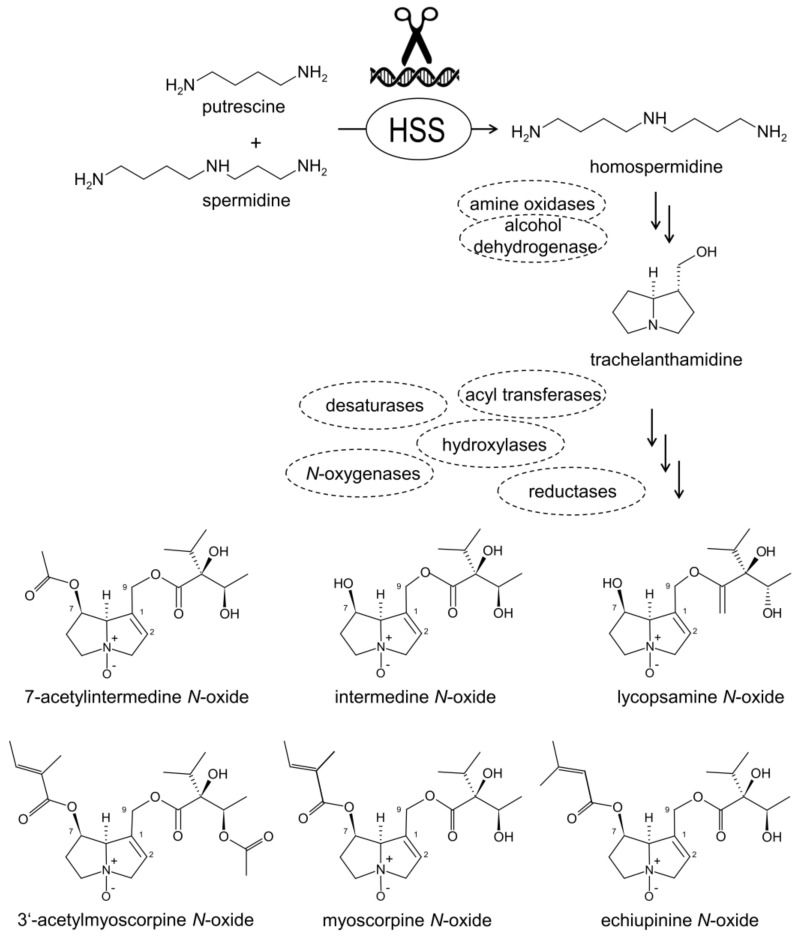
Pyrrolizidine alkaloid (PA) biosynthesis. Homospermidine synthase (HSS) catalyzes the formation of homospermidine as the exclusive substrate for PAs. Further enzymes are postulated (labeled by dashed ovals), but the sequence of enzymatic conversions is not known. Structures of the intermediate trachelanthamidine and of six PAs characteristic for hairy roots (HRs) of *S. officinale* are shown in their *N*-oxide form.

PAs are specific plant secondary metabolites that are constitutively produced by plants as part of their natural chemical defense against herbivores [16,17]. They occur scattered in about 3% of all plants world-wide and frequently in species relevant for human and animal consumption [18,19,20]. Thirteen distantly related angiosperm plant families have been reported to contain PA-producing species with the main occurrences in the Boraginaceae, Asteraceae, Apocynaceae, Convolvulaceae, Orchidaceae, and Fabaceae [21,22]. Over 660 PAs have been identified in about 6000 plant species and about half of them are deemed toxic [23]. Structurally, PAs contain either a saturated or a 1,2-unsaturated necine base moiety that is esterified with one or more necic acids. PAs with the unsaturated necine base are regarded as potentially toxic exerting hepatotoxic, pneumotoxic, hemolytic, antimitotic, teratogenic, mutagenic, and carcinogenic effects for, both, humans and livestock [14,16,24]. A deeper understanding of PA biosynthesis should allow the development of strategies of metabolic engineering for the manipulation of the PA biosynthetic pathway in order to remove these toxic metabolites and to allow the use of the other beneficial natural products in these medicinal plants. According to our present knowledge, the first step in PA biosynthesis is catalyzed by homospermidine synthase (HSS) [25,26]. HSS has evolved several times independently in several PA-producing lineages by the duplication of the gene encoding deoxyhypusine synthase (DHS) [27,28,29], an enzyme of primary metabolism that is involved in the posttranslational activation of the eukaryotic translation initiation factor 5A [30]. HSS catalyzes the transfer of the aminobutyl group from spermidine to putrescine resulting in the PA-specific precursor homospermidine (Figure 1) [25,31]. Unlike its substrates, namely putrescine and spermidine, homospermidine is regarded as a rare polyamine that is a characteristic intermediate in PA-producing plants, as it is exclusively incorporated into the necine base backbone [32]. HSS has been shown to be expressed in diverse specialized cells and tissues in various plant lineages [33,34,35]. In comfrey, HSS has been localized in the cells of the root endodermis and in specific cells of young leaves subtending a developing inflorescence [36,37]. The identification and characterization of further enzymes involved in PA biosynthesis and strategies for their manipulation by the most recent genome-editing techniques might pave the way for the development of a new generation of medicinal plants with a reduced level or even the absence of toxic PAs to provide raw materials for safer phytopharmaceuticals.

Over the past few decades, various approaches with a wide range of aims have been used to induce gene silencing in plants. These objectives include a better understanding of gene functions, an improvement in the responsiveness of plants to abiotic and biotic stresses, and the fine-tuning of metabolic pathways in order to influence the biocatalysis of bioactive natural products [38,39,40,41] through various technologies based on targeting either RNA or DNA with the goal of being able to affect gene expression and protein function [42]. RNA-targeting methods—such as antisense RNA, RNA interference (RNAi), and virus-induced gene silencing (VIGS)—have been successfully conducted in a wide number of plants in order not only to investigate the functions and physiological significance of certain genes, but also, quite recently, to manipulate PA levels [43]. However, the silencing effects implied by these approaches often merely show a transient reduction in gene expression (knock-down) and sometimes unexpected effects attributable to self-genetic feedback regulation [44,45]. A more efficient method has been the introduction of DNA-targeting systems such as transcription activator-like effector nucleases (TALENs), zinc-finger nucleases (ZFNs), and—more recently—clustered regularly interspaced short palindromic repeats (CRISPR)/Cas9 as efficient genome-editing techniques for the complete and persistent loss of gene function [46,47]. CRISPR/Cas9 technology, which was considered as the breakthrough of the year 2015 [48] and was honored by the Nobel Prize in 2020 [49], is based on the ability of the Cas9 enzyme, a RNA-guided endonuclease from *Streptococcus pyogenes*, to introduce a double-strand break into DNA at a targeted location. A complementary single-guide RNA (sgRNA) fused to Cas9 enables the use of specific recognition sequences of 20 nucleotides in length that bind to the target DNA region at a point at which Cas9 will then cut three nucleotides upstream of a DNA motif called the protospacer adjacent motif (PAM). The sequence of the PAM varies according to the origin of the Cas9 endonuclease [50]. Today, CRISPR/Cas9 is regarded as the best choice for gene editing and targeted mutagenesis in numerous plants [51,52,53,54]. The advantage of creating null mutations in the T0 generation is particularly advantageous for long-term studies or economical and industrial applications.

Here, we report the successful CRISPR/Cas9-mediated knock-out of the HSS-encoding gene in *S. officinale* by using hairy roots (HRs) generated by stable transformation with the soil-borne *Agrobacterium rhizogenes*. We show, by genotyping, HPLC-based polyamine analysis, and GC-MS-based PA analytics, that the non-functionalization of HSS results in a drastic reduction of homospermidine and a complete elimination PAs in the resulting HRs. These results are decisive in planta evidence for the initiation of PA biosynthesis by HSS and are consistent with a previous knock-down study of comfrey conducted by our group using an RNAi approach [43]. Based on these findings, we show, by using HRs as an in vitro system, the possibility of employing the knocking-out of HSS in PA-producing plants in order to develop PA-free plant material that will, in turn, allow its use for medicinal preparations without safety concerns. Furthermore, we will discuss the impact of these PA-free plants on further studies on the characterization of the complete pathway of PAs.

## 2. Results

**Figure 2:**
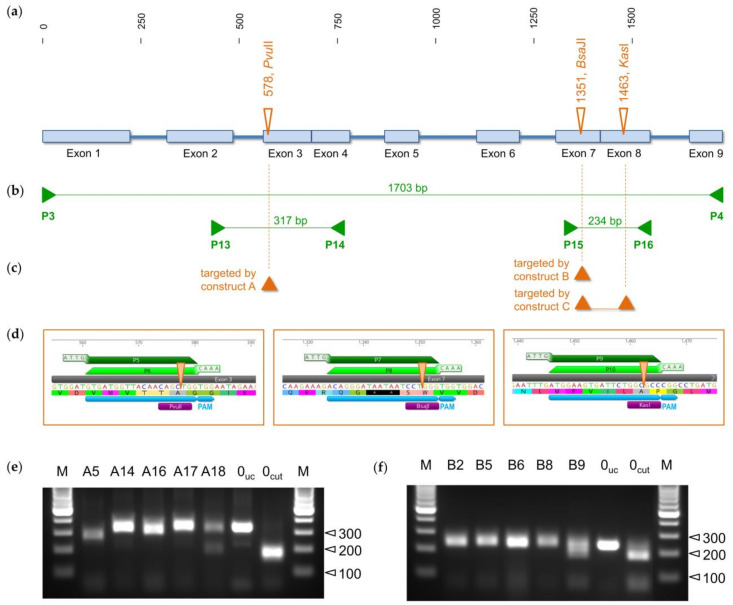
CRISPR/Cas9 approach for the manipulation of the *hss* gene. (**a**) Genomic structure of the *hss* gene with restriction sites at exons 3, 7, and 8 that were targeted by sgRNAs with specific protospacer motives. (**b**) Primer positions for the amplification of the complete and partial gene sections. (**c**) Gene regions targeted by the CRISPR/Cas9 constructs A, B, and C. (**d**) Protospacer sequences used in this study targeting exon 3, exon 7, and exon 8. The predicted sites of the Cas9-mediated double-strand breaks within the recognition sites of the endonucleases are indicated by arrow heads. Primers used for the sgRNA cassette are given in Table A1. PAM, protospacer adjacent motif. (**e**,**f**) Restriction analyses of genomic fragments amplified with primer pairs P13/P14 including exon 3 and P15/P16 covering exon 7 and 8 after incubation with *Pvu*II (**e**) and *Bsa*JI (**f**). Resulting fragments were separated on a 3% agarose gel by using the GeneRuler 100 bp DNA ladder.

In order to implement the CRISPR/Cas9 technology in comfrey, we amplified the complete *hss* gene to identify intron positions. Intron positions of HSS- and DHS-coding genes are known to be highly conserved with respect to position and phase, even when they are compared with the DHS of humans. Based on such an alignment, we mapped, on the 1703 bp-long gene, nine exons of which exon 3/exon 4 and exon 7/exon 8 are not separated by introns in *S. officinale* (Figure 2a). In order to edit this genomic sequence, we used the protocol and the plasmids provided by the group of Holger Puchta (Karlsruhe Institute of Technology, Germany, [55]). We generated binary vector constructs harboring the Cas9 protein expression cassette, the gene encoding hygromycin B phosphotransferase to allow selection of transgenic plants by hygromycin, and the sgRNAs targeting the *hss* gene. Three different sequence stretches within the exons 3, 7, and 8 were used to design N_20_ oligomers for the sgRNAs. The two sgRNAs targeting exon 3 and exon 7 resulted in constructs A and B, respectively. In addition, one construct, namely construct C, contained two different sgRNAs, one designed to target exon 7 and the other targeting exon 8 (Figure 2c). Using these binary vector constructs, we generated HRs of *S. officinale* by transformation with *A. rhizogenes*. Transgenic HRs were used to extract genomic DNA and to amplify, via the polymerase chain reaction (PCR), the targeted gene regions. Restriction enzyme analysis initially screened the mutated HR lines and was followed by the sequencing of the corresponding regions to reveal modifications resulting from the gene-editing event in the *hss* gene.

### 2.1. Restriction Analysis as a Screening Strategy to Identify HR Lines with Modified Genomic Sequence

During the design of the N_20_ motives for the sgRNAs, regions of the genomic DNA were selected that possessed restriction sites positioned three bases upstream of the PAM (5′-NGG-3′). The idea was to simplify the screening for transgenic HR lines that were modified at the position that was targeted by the CRISPR/Cas9 complexes. Such a modification within the sequence motif of an endonuclease should result in the inability to cut the genomic DNA at this position with the respective enzyme. Such a screening approach enables the number of lines with a putatively modified *hss* gene to be reduced in an early experimental step. In this study, the recognition sites for the endonucleases *Pvu*II, *Bsa*JI, and *Kas*I were part of the target sites within exons 3, 7, and 8, respectively (Figure 2a,d). After transformation with *A. rhizogenes* and selection of transgenic lines by growth on a hygromycin-containing medium, genomic DNA was used for PCR amplification of the genomic regions of exon 2 to 4 and exon 7 and 8 resulting in amplicons of 317 bp and 234 bp, respectively (Figure 2b). As phenolics present in *S. officinale* might have an impact on the quality of genomic DNA and, therefore, on the PCR amplification, polyvinylpyrrolidone was added to the buffer during genomic DNA extraction. Figure 2e,f show the PCR fragments obtained from the DNA of selected HRs after digestion with the endonucleases *Pvu*II and *Bsa*JI, respectively, following separation via an agarose gel. For comparison, the PCR fragment obtained from a control line resulting from transformation with the empty vector was included without digestion (HR 0_uc_) and after digestion with the respective endonuclease (HR 0_cut_). Of the analyzed HRs resulting from transformation with vector construct A, no cuts were detectable for HR-A14 and HR-A17, an uncompleted cut was obtained for HR-A18, and a prominent and a slight size shift were observed for HR-A5 and HR-A16, respectively (Figure 2e). For the HRs resulting from transformation with construct B, no cuts are detectable for lines HR-B2, HR-B5, HR-B6, or HR-B8, while the fragment of line HR-B9 seemed to be cut partially (Figure 2f). These observations suggested that all of the analyzed HRs had some modifications within the amplified genomic sequence. The reason for using construct C, which harbored two sgRNA targeting specific sites in exon 7 and 8, was to introduce two simultaneous double-strand breaks in order to facilitate a sequence-specific deletion of about 112 bp. Analysis of PCR amplicons covering the genomic DNA containing exon 7 and exon 8 should have allowed the straightforward identification of lines with large fragmental mutations resulting in a nonfunctional *hss* gene. However, none of the six tested lines from the transformation with construct C showed any size shift after agarose gel electrophoresis (data not shown).

### 2.2. Characterization of Mutations within the hss Gene of Transgenic HR Lines by Genotyping

In addition to the restriction enzyme analysis, PCR amplicons of selected HR lines were sequenced. The control lines generated by transformation with an empty vector control, i.e., the binary vector still containing the *ccdB* suicide gene, were analyzed to verify that they did not contain any mutations within the targeted exons of the *hss* gene. Then, we aligned the sequences of these control lines with those amplified from the HR lines resulting from transformation with one of the three constructs containing the expression cassettes for the specific sgRNAs targeting the *hss* gene. Table 1 summarizes the results of the CRISPR/Cas9-editing approach for the target sites of exon 3 and exon 7. In addition, the analyzed HRs were classified into three groups with respect to the expected effects on *hss* gene function: (i) putative knockout (KO) if both alleles were altered and no WT allele was detected; (ii) putative knockdown (KD) if one allele was affected, but a WT allele was still present; and (iii) putative non-edited (NE) if only WT alleles could be identified (Table 1). Except for HR-A3, all analyzed HRs resulting from transformation with construct A showed modifications of the genomic sequence within the target site. Most modifications were small deletions ranging between 2 to 16 nucleotides in length, of which the 16 bp deletion was large enough to be detectable by electrophoretic separation (Figure 2e). For five out of nine tested HR lines, the analyzed sequence was identical in both alleles (‘homozygous’ mutation type). One line, HR-A5, showed different mutations within the two alleles, i.e., deletions of 52 and 78 nucleotides, respectively (‘biallelic’ mutation type). Both HR-A17 and HR-A18 exhibited an identical 3 bp deletion but also possessed the wild-type sequence, indicating that only one of the two alleles of the *hss* gene had been edited (‘monoallelic’ mutation type). This was also observed for all seven tested HR lines resulting from transformation with construct B, as we found the wild-type allele, in addition to the modifications within the target region of exon 7, in these HRs. Genotyping of selected HR lines that resulted from transformation with construct C established that they had not been modified at the motif in exon 8 (data not shown). Instead, we identified three out of six tested lines with monoallelic exon 7 mutations that were similar to those detected in lines resulting from transformation with construct B (data not shown). These data suggested that the sgRNA targeting exon 8 might have been less efficient under our experimental setup. Therefore, the HR lines resulting from transformation with construct C were eliminated from further analyses.

### 2.3. Chemotyping of Transgenic HR Lines Reveals Effects of CRISPR/Cas9 Editing on Levels of Homospermidine and PAs

Deletions or insertions of numbers of nucleotides that are not a multiple of three result in a frame-shift of the downstream sequence and, hence, a completely functionless *hss* gene. The consequence of larger deletions will be the same, as the amino acid sequence of HSS is highly conserved suggesting that such large deletions (up to 78 bp encoding 26 amino acids in HR-A5) will affect functional relevant residues. For deletions of 3 or 9 nt that have been detected in several HR lines resulting from transformation with construct A (HR-A14, HR-A17, HR-A18) and construct B (HR-B8) and that do not cause a frame-shift in the downstream sequence, functional analyses need to show whether the changes in the amino acid sequence are sufficient to have an effect on HSS activity. Any reduced activity or the inactivation of HSS should have detectable effects on the amount of homospermidine and of PAs, for which homospermidine is the precursor.

**Figure 3:**
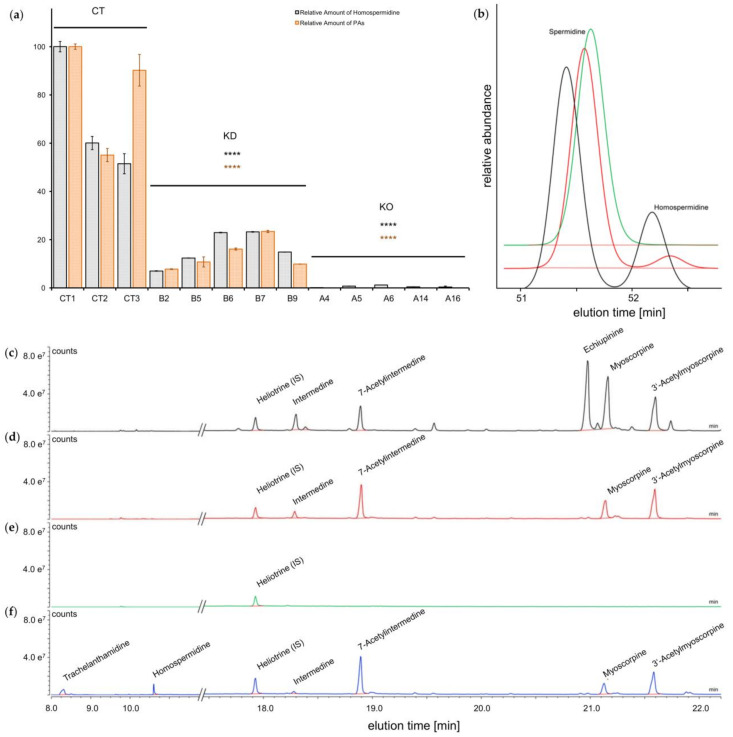
Chemotyping of transgenic HR lines. Homospermidine and PA levels (**a**) in HR lines resulting from transformation with the empty vector (control lines, CT) and the CRISPR/Cas9 constructs A and B. Classification as knock-down (KD) and knock-out (KO) lines follows the prediction according Table 1. Relative intensities have been calculated in relation to the internal standards (diaminoheptane and heliotrine) and the amount of extracted biomass (fresh weight and dry weight) for quantification of homospermidine and PAs, respectively. 100% values correspond to 1961 pmol/g fresh weight homospermidine and 8084 µg PA per g dry weight. Error bars represent the standard error of the mean (SEM) of three technical replicates of the same line. Four asterisks indicate a highly significant downregulation in comparison with the control lines (*p* < 0.0001). (**b**) Comparison of polyamine levels of a control line (HR-CT1, black), a knock-down line (HR-B7, red), and a knock-out line (HR-A14, green). A HPLC chromatogram of all detected polyamines is shown in Figure A1. (**c**) to (**f**) Total ion chromatograms of purified extracts of selected HR lines: control line (HR-CT1) (**c**), knock-down line (HR-B7) (**d**), knock-out line (HR-A14) (**e**), and knock-out line (HR-A14) fed for four weeks with homospermidine (**f**). A complete total ion chromatogram of control line HR-CT1 is shown in Figure A2.

In order to test the effect of the CRISPR/Cas9 gene-editing event observed in several of the generated HRs, homospermidine was quantified in several edited lines resulting from construct A and construct B in comparison with the control lines by HPLC-based polyamine analyses (Figure 3a,b). In comparison with the homospermidine level of the control lines resulting from transformation with the empty vector (average = 1382 pmol/g FW), the HRs predicted to be knock-down lines (Table 1, ‘KD’) did indeed show a drastic reduction of homospermidine levels by approx. 80% (average = 315 pmol/g FW), most likely because of the inactivation of the *hss* gene on only one of the two alleles. The HRs predicted to be knock-out lines (Table 1, ‘KO’) because of the detection of mutations in both alleles were almost free of homospermidine (average = 11 pmol/g FW). The reduction in both the predicted downregulated and knock-out lines was highly significant (*p* < 0.0001) supporting the predictions of the gene-editing effects on the *hss* gene in Table 1. Compared with the control lines, the mutated lines with the drastically reduced homospermidine levels showed no visible differences in growth or development indicating that homospermidine is not required for primary metabolism but is most likely only needed for PA biosynthesis. Furthermore, the level of the other amines included in the polyamine analyses (e.g., putrescine, spermidine) was stable in all analyzed HR lines, irrespective of whether they were control lines or lines with downregulated homospermidine levels. This suggests that the polyamine pool of primary metabolism is independent of the pool of accumulating homospermidine.

As homospermidine is regarded as the first specific intermediate of PA biosynthesis, we tested whether the effect of the mutations on the homospermidine level correlated with the level of PAs. Using GC-MS for PA quantification, we were able to demonstrate that the HR lines classified as knock-down lines showed a reduction of the detectable PAs by approx. 80% (average = 1101 µg/g DW) in comparison with the control lines (average = 6608 µg/g DW). In the knock-out lines, no PAs were detectable (even as traces), in contrast to the homospermidine analyses. Figure 3 shows the total ion chromatograms of an extract of a control line (HR-CT1, Figure 3c), a downregulated line (HR-B7, Figure 3d), and a knock-out line (HR-A14, Figure 3f). PAs detectable in the control lines were identical to those found in wild-type roots of *S. officinale* [56] and in previous analyses of HRs [43] with respect to intermedine, 7-actylintermedine, echiupinine, myoscorpine, and 3′-acetylmyoscorpine as the main PAs. The chromatograms of the knock-down lines revealed reduced PA levels and a modified PA pattern in which echiupinine, the major PA, and some minor PAs were absent. In the chromatogram of the knock-out line, only the internal standard was detectable. Of note, the deletion of three amino acids in the knock-out line HR-A14 without a frame-shift was sufficient to inactivate HSS completely.

### 2.4. Recovery of PAs in HRs with a Defective hss Gene by Feeding of Homospermidine

To test whether the absence of PAs in the knock-out line was merely attributable to the inactivation of the gene encoding HSS and not to any other side effects of the CRISPR/Cas9 gene-editing approach, we fed the knock-out line HR-A14 with homospermidine for four weeks. Figure 3f shows that, in this HR line, the PAs were detectable once again, with a pattern similar to that of the knock-down lines, i.e., without a peak for echiupinine, but with additional peaks for homospermidine and trachelanthamidine.

## 3. Discussion

Plants are well-known to produce a huge diversity of metabolites that are part of their secondary metabolism and that have various ecological functions [57]. Many of these secondary metabolites (also called ‘specialized metabolites’) are important pharmaceuticals because of their biological activity, whereas others are problematic for human use because of their toxicity. If beneficial and toxic compounds co-occur in a plant, its use as a phytopharmaceutical is problematic [3,58]. Sophisticated extraction and purification techniques can help to overcome some of these disadvantageous. Most recent strategies of metabolic engineering focus on the manipulation of pathways to increase yield or on the transfer of complete biosynthetic routes into a heterologous system to optimize handling, harvest, and yield [59,60,61,62,63].

The pharmaceutical application of *S. officinale*, a traditional medicinal plant, is restricted because of the occurrence of PAs. One target that might be used to influence PA-levels is the HSS that has been identified in previous in vitro studies as being the first specific enzyme of PA biosynthesis [25,32]. A recent in planta study generating a RNAi-mediated knockdown in our group has shown that the downregulation of *hss* transcript levels correlates with a significant decrease in both homospermidine and PA levels and showed the potential of HRs to manipulate PA levels as PA biosynthesis is localized in the roots [43]. Based on these findings we decided to test the revolutionary CRISPR/Cas9 technology as a more straightforward approach for obtaining HR lines with a permanent and stably inactive HSS to overcome disadvantages associated with the RNAi technique. Such disadvantages include the variation in the silencing efficiency [64] and the often variable levels of secondary metabolites in the control lines. Such effects are attributed to the agrobacterial *rolB* gene that is transferred to the plant genome and that might influence the activity of transcription factors [65].

Use of the CRISPR/Cas9 technology might result in the efficiency of sgRNAs in targeting the CRISPR-associated Cas9 to the gene of interest varying in dependence on the protospacer sequence [66]. Therefore, we have included, in our experimental approach, several tools for the fast and efficient screening of transgenic HRs based on the analysis of the size of PCR amplicons in order to detect larger insertions or deletions and to test for inactivated recognition sites for selected endonucleases. All these screening tools have their pros and cons. Whereas restriction-based screening limits the number of suitable protospacer sequences dramatically, the strategy based on the simultaneous use of two sgRNAs (construct C) requires the good efficiency of both sgRNAs in guiding the CRISPR-associated Cas9 to the targeting sequence. However, such screening tools might facilitate the screening in those cases in which gene-editing events are rare. The data presented here show that 8 out of 9 tested HRs resulting from transformation with construct A targeting exon 3 exhibit editing effects superseding the need for an extensive screen of a large number of roots. Therefore, the number of HRs tested in this study remains too small for a comparison of the efficiency of the three tested constructs or for testing the predictability of our screening tools extensively.

Table 1 presents the modifications that resulted from the genome editing of the *hss* gene. In no case have we detected base replacements. Instead, we have determined mainly deletions with a length between 1 nt and 78 nt and a few insertions of 1 nt in length. Many of these are identical on the two alleles, suggesting that one allele is edited first and then serves as a template for the repair of the second allele. However, in HR-A5, we have observed two different mutations (deletion of 52 bp and 78 bp) on the two alleles. In two out of nine tested HRs resulting from transformation with construct A and in all HRs resulting from transformation with construct B, editing events were only detectable in one of the alleles. We have confirmed that the second allele represents the unmodified sequence of a functional *hss* gene after cloning of the PCR amplicons by sequencing. Polyamine and PA analyses show that this ‘wild-type-like’ allele is sufficient for the occurrence of homospermidine and PAs, although at a significant lower level in comparison with the control lines. HR lines with mutations in both alleles of the *hss* gene are completely free of PAs, confirming that HSS is a central enzyme of PA biosynthesis and that it is encoded by a single gene whose activity cannot be restored, even in part, by other enzymes of the plant. The observation that knock-out HRs are able to produce PAs if they are fed with homospermidine confirms that only the inactivation of HSS is responsible for the PA-free phenotype, and that no other unidentified off-target effects of the CRISPR/Cas9 approach are involved.

In contrast to the PAs that are undetectable in the knock-out lines, we have still found traces of homospermidine in these HRs. In earlier studies, traces of homospermidine have been detected in many plant lineages, irrespective of whether these plant species produce PAs [67]. This occurrence has been attributed to ubiquitous DHS that has been shown previously to be expressed in all plant tissues and that is able to catalyze the formation of homospermidine under in vitro conditions, most likely as a side reaction with no relevance in vivo [30]. Our data suggest that, indeed, DHS may contribute—even though only in part—to the homospermidine pool of the plant. As this homospermidine is not incorporated into PAs, this pool has to be well separated from the homospermidine pool feeding PA biosynthesis. A further argument for such a compartmentalization between the polyamine pool of primary metabolism and the homospermidine pool for PA biosynthesis is the observation that, in our HR lines, we have not observed increased levels of spermidine that might compensate for the reduced homospermidine levels. Such compensation has been seen earlier in tobacco plants, in which the *hss* gene of *Senecio vernalis* (Asteraceae) is expressed under the control of the 35S promoter. Here, homospermidine accumulates at the expense of spermidine, whose levels are reduced by about 80% [68]. The latter authors have shown that the total amount of the two triamines, namely spermidine and homospermidine, remain constant in comparison to those of control plants, suggesting that homospermidine can functionally replace spermidine in primary metabolism, and that the total triamine concentration is properly regulated. Moreover, inhibitor studies with *Senecio* roots have shown that endogenously formed homospermidine is exclusively incorporated into PAs and is well separated from the highly dynamic polyamine pool of primary metabolism [32]. The subcellular compartmentalization and trafficking of secondary metabolites and intermediates in plants is a well-known phenomenon [69,70,71,72,73] and is obviously also of relevance for PA biosynthesis.

Earlier experiments have shown that the silencing of HSS by RNAi results in reduced PA levels, suggesting that the total amount of PAs is dependent on the amount of transcript [43]. This interpretation is supported by our HR lines that have only one functional allele of the *hss* gene and that also exhibit significantly reduced levels of homospermidine and PAs. Obviously, PA biosynthesis is dosage dependent, i.e., two functional alleles result in more transcript, more enzyme, and more product than only one functional allele. This observation suggests that PA biosynthesis is, at least not exclusively, regulated by transcription factors as has been shown for other classes of secondary metabolites [74,75,76]. Another interesting observation is that these HR lines with only one functional *hss* allele do not have the same PA pattern as the control lines. The most prominent PA, echiupinine, and some minor PAs are missing in the PA bouquet of these lines suggesting that a later step in PA biosynthesis is affected by the knock-out of HSS. Reduced levels of pathway intermediates might be insufficient to induce certain enzymatic conversions of the pathway. Of note, the PA pattern of the knock-out lines fed with homospermidine is also more similar to that of the knock-down lines than to that of the control lines, with the exception that, in the fed knock-out lines, homospermidine and the early intermediate trachelanthamidine are detectable.

The PA-free HR lines resulting from this project represent not only a feasibility study for CRISPR/Cas9-mediated editing in comfrey but offer several economically and scientifically important options. Economically, such HRs can easily be scaled up as an in vitro system for the rapid and massive production of valuable pharmaceuticals [77]. In comfrey, mainly phenolics such as rosmarinic acid and allantoin are regarded as the active principle for their main therapeutic applications [12]. The identification of lines with high levels of these compounds for economic use should be possible within our PA-free HR lines. Furthermore, plants can be regenerated from HRs for cultivation as a raw material for phytopharmaceuticals with no safety concerns because of the putative content of PAs. However, if such plants are regenerated from our HR lines, they will then be classified as genetically modified organisms as their genomes contain the expression cassettes for the Cas9, the sgRNA, and the hygromycin resistance gene and result from a CRISPR/Cas9-mediated editing approach. Scientifically, the PA-free HRs offer a promising tool for the identification of potential intermediates of PA biosynthesis as these are completely absent in knock-out HR lines. Feeding of these HRs with homospermidine should allow the detection of at least some of the intermediates of PA biosynthesis by a comparative approach using unfed lines as control. Based on such data, the sequence of further postulated biosynthetic steps involving amine oxidases, reductases, desaturases, acyl transferases, *N*-oxygenases, or hydroxylases might be elucidated [78,79]. Furthermore, as the identification of the genes encoding enzymes relevant for PA biosynthesis is often hampered by the lack of in planta evidence in these “non-model” species [80], this study shows the potential of CRISPR/Cas9-based approaches for testing such candidates for the functional relevance on PA biosynthesis.

## 4. Materials and Methods

### 4.1. Plant Material

Seeds of *S. officinale* (Rühlemann’s Kräuter & Duftpflanzen) were grown in the greenhouse of Kiel Botanic Gardens at approx. 23 °C/18 °C day/night cycles in pots containing a mixture of potting soil (TKS2, Floragard) and lava granulate at a ratio of 3:1 from April to September. An herbarium specimen was deposited in the Herbarium KIEL with the accession number KIEL0005102.

### 4.2. Cloning of sgRNAs and Generation of Binary Vector Constructs for Plant Transformation

For generation of the CRISPR/Cas9 vector constructs, the following plasmids were obtained from the Puchta group (Karlsruhe Institute of Technology, [55]):

pEn-Chimera as entry vector with ampicillin resistance and containing the sgRNA expression system with the *Arabidopsis* U6-26 promoter [81,82] flanked by attR sites for Gateway cloning into the binary destination vector.

pEn-C1.1 as entry vector with properties identical to that of pEn-Chimera except for additional *Bsu*36I and *Mlu*I restriction sites between the sgRNA expression cassette and attR sites to allow the insertion of a second sgRNA expression cassette into the binary destination vector by a Gateway-independent cloning strategy.

pDe-CAS9 as binary plasmid for *Agrobacterium*-mediated transformation harboring, in the T-DNA, the Cas9 (of *S. pyogenes*) expression cassette, a resistance gene against phosphinothricin for selection of transgenic plants, and the *ccdB* suicide gene flanked by attR sites for integration of the sgRNA expression cassette from the pEn-Chimera entry vector by Gateway cloning. In addition, this vector contains *Bsu*36I and *Mlu*I restriction sites to allow the integration of a second sgRNA expression cassette from the pEn-C1.1 vector. The backbone provides spectinomycin resistance for propagation in *E. coli*.

To allow the selection of transgenic HRs of *S. officinale* on hygromycin, the hygromycin resistance cassette from the vector pH7WG2D,1 [83] was amplified using primer pair P1/P2. The resulting PCR product of 1807 bp was digested with *Hind*III and cloned into the *Hind*III-linearized pDe-CAS9 to replace the phosphinothricin resistance cassette. This vector was named pDe-Cas9-HYG. All primer sequences are presented in Table A1.

The gene encoding HSS of *S. officinale* was identified by amplification with primers previously used for amplification of the respective cDNA (P3/P4, [27]) followed by cloning and sequencing. This genomic sequence (Acc. no. MW365954) was compared with the cDNA (Acc. no. AJ704851) to identify intron positions in order to avoid inoperable protospacers corresponding to a cDNA sequence motif that might be interrupted by an intron at the genomic level. For the prediction of potential CRISPR sites, we used the software Geneious Prime (V2020.1.2, Biomatters, Auckland, New Zealand). We selected the protospacer sequences for editing *hss* of *S. officinale* by employing the following guidelines: (i) Each specific protospacer sequence comprised 20 bp target sequence preceding a protospacer adjacent motif (PAM) of *S. pyogenes* Cas9 (NGG) to direct the Cas9 enzyme to the cleavage site; (ii) the double-strand break introduced by Cas9 had to affect the exonic sequence in order to ensure that the induced frame-shift mutations would have functional consequences; (iii) because of the close similarity of the coding region of *hss* and its paralog encoding DHS, the protospacer sequence had to be sufficiently specific to target only the HSS-encoding gene, and not the essential DHS-coding gene; (iv) in order to simplify the later screening of transgenic lines harboring a modification that had resulted from the editing event, we selected protospacer motives in which the Cas9 cleavage site lay within the recognition site of a restriction enzyme in order to ensure that PCR amplicons covering this region of the *hss* gene would only be cut by the restriction enzymes if they remained unmodified.

By carrying out this strategy, three protospacers targeting the three different exons (i.e., exons 3, 7, and 8) (Figure 2d) were selected for cloning of the sgRNA. We followed the strategy described by Schiml et al. [55] by designing complementary oliogonucleotides with appropriate overhangs for cloning into the *Bbs*I-linearized entry vectors pEn-Chimera (protospacers for exon 3 and exon 7, primer pairs P5/P6 and P7/P8, respectively) and pEn-C1.1 (protospacer for exon 8, primer pair P9/P10). The sgRNA cassettes from the resulting pEn-Chimera constructs for exon 3 and exon 7 were transferred by Gateway cloning into the destination vector pDe-Cas9-HYG by using LR clonase II (Thermo Fisher Scientific, Waltham, MA, USA) resulting in construct A (targeting exon 3) and construct B (targeting exon 7). To prepare construct C harboring two sgRNA cassettes (targeting exon 7 and exon 8), the sgRNA cassette targeting exon 8 was subcloned from pEn-C1.1 to construct B by using *Mlu*I digestion [55]. The resulting binary vector constructs A, B, and C were propagated in *E. coli* TOP10 cells before the success of the cloning was confirmed by sequencing with primer pair P11/P12 that amplified the regions covering the Gateway and the *Mlu*I cloning sites (Eurofins Genomics, Ebersberg, Germany).

### 4.3. Transformation of A. Rhizogenes and Generation of Transgenic HRs

Binary vectors were transformed into cells of the electrocompetent *A. rhizogenes* strain ATCC 15,834 (ATCC, Manassas, VA, USA) [84] according to the method described by Wise et al. [85]. Briefly, the electrocompetent cells were thawed on ice immediately before use, and a 40 µl aliquot was mixed with 500 ng plasmid DNA in ddH_2_O in an ice-cold 1.5-mL tube and gently mixed by tapping the tube several times. The mixture was transferred subsequently to an ice-cold microelectroporation cuvette and incubated for an additional 30 min on ice. The cuvette was inserted into an electroporator (BioRad, Hercules, CA, USA), and transformation was performed with a pulse of 2.5 kV/cm for about 6 ms. For the empty vector control, we used the pDe-Cas9-HYG vector, as the *ccdB* suicide gene does not affect Agrobacteria. For infection of young leaves of *S. officinale*, the Agrobacteria harboring the binary plasmids were used as described previously [43] with the following modification: instead of incubating the leaf explants with the *Agrobacterium*-containing medium under agitation, the *A. rhizogenes* cells were harvested by centrifugation, resuspended in liquid MS20 medium (hormone-free MS medium with 20% of the original amount of NH_4_NO_3_ [86]), and used for infection by gently scratching the main and lateral veins on the lower epidermal surface of the leaves before transference of the leaves upside down to solid MS20 media (1%, w/v, agar) in order to avoid the prominent growth of the bacterial cells on the medium. Developing HRs were cut from the leaf and transferred to fresh MS20 plates containing 250 µg/mL of a mixture of ticarcillin and clavulanic acid (mixed at a ratio of 15:1, Duchefa, Haarlem, Netherlands) and hygromycin B at a concentration of 25 µg/mL. Transgenic HR lines that survived selection on hygromycin-B-containing medium were grown in darkness at 16 °C on solid MS20 medium in a climate chamber and were used later for genomic analysis and the quantification of homospermidine and PA levels. Of note, not transgenic roots do not survive on hygromycin-containing medium. Hygromycin-resistant HRs were tested for the successful integration of the *rolA* gene. The absence of remaining agrobacteria was tested by amplification of the *virD* gene [43] (Figure S1). To yield enough root material for PA analysis, liquid cultures in MS20 medium (supplemented with ticarcillin/clavulanic acid to prevent the growth of both agrobacteria and other contaminating microorganisms) were inoculated from a well-developed HR line and cultured in the dark at 16 °C for 14 d at 130 rpm on an orbital shaker (Infors, Bottmingen, Switzerland). After harvest, the HRs were washed with water, dabbed dry with tissue paper, and freeze-dried.

### 4.4. Analysis of HR Lines for Mutations within the hss Gene

Approximately 20 mg of each of the transgenic HR lines growing on MS20 medium plates were taken for the extraction of genomic DNA by the NucleoSpin Plant II kit (Macherey–Nagel, Germany) according to the manufacturer’s instructions with one modification: to improve the quality of DNA, approx. 10 mg polyvinylpyrrolidne (Polyclar AT, Serva, Heidelberg, Germany) was added to lysis buffer PL1 to bind the phenolics present in roots of *S. officinale*. The quality and concentration of the genomic DNA were analyzed by a NanoDrop2000 UV–vis Spectrophotometer (Thermo Fisher Scientific). To amplify the regions of genomic DNA targeted by our CRISPR/Cas9 approach, primer pair P13/P14 was used for HRs resulting from transformation with construct A (targeting exon 3) and primer pair P15/P16 for HRs resulting from transformations with construct B (targeting exon 7) and construct C (targeting exon 7 and exon 8) (Figure 2b). Phusion DNA polymerase (Thermo Fisher Scientific) was used as a proofreading enzyme in reactions of 25 µl with 10 ng genomic DNA as template. PCR amplicons were purified with the NucleoSpin^®^ Gel and PCR Clean-up kit (Macherey&Nagel, Germany) and used for restriction analysis with the endonucleases *Pvu*II and *Bse*DI (isoschizomer to *Bsa*JI) for the characterization of HRs resulting from transformation with constructs A and B, respectively, and with *Bse*DI and *Kas*I for HRs resulting from transformation with construct C. The size of the resulting fragments was estimated after separation by 3% (*w*/*v*) agarose TBE gel-electrophoresis together with the GeneRuler 100 bp DNA ladder (Thermo Fisher Scientific).

For sequencing, the amplicons were sent out directly after purification with the NucleoSpin^®^ Gel and PCR Clean-up kit (Macherey&Nagel) according to the manufacturer’s instructions or were subcloned into the pGEM-T Easy vector system (Promega, Fitchburg, WI, USA) after addition of an ‘A-tail’ by incubation with GoTaq DNA polymerase (Promega). Subcloning was necessary when direct sequencing resulted in ambiguous sequence signals attributable to the presence of two differentially edited alleles. Plasmid DNA of up to five colonies resulting from each cloning reaction (selected after blue/white screening) was purified and sequenced to identify the sequences of the individual alleles. The genomic sequences were aligned and analyzed by Geneious software with the “map to reference” and “pairwise alignment’’ tools.

### 4.5. Extraction and Quantification of Homospermidine

**Figure A1:**
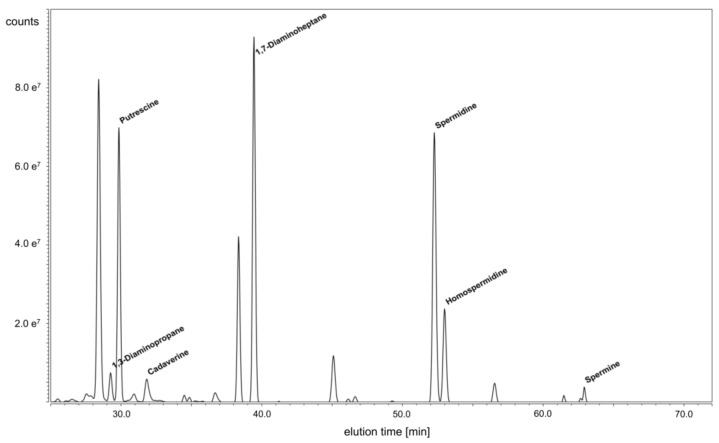
HPLC chromatogram of polyamines of the hairy root control line HR-CT1. All labeled peaks were identified by running pure standards under the same experimental conditions.

Fresh material (200 mg) from selected transgenic HRs grown on solid MS20 medium was transferred to 2-mL reaction tubes (Sarstedt, Nümbrecht, Germany) and powdered by means of metal balls in liquid nitrogen in a Mixer Mill MM 400 (Retsch, Haan, Germany) at a frequency of 30/s for 1 min. The powder was extracted in 1 mL perchloric acid (5%, *v*/*v*) containing 2 nmol 1,7-diaminoheptane as an internal standard under freeze-thaw cycles according to Minocha et al. [87]. Subsequently, the pH was raised to pH 9–10 with 10 M NaOH (approx. 50 μL per 1000 μL extract). Precolumn derivatization and chromatography of polyamines were carried out according to Kaltenegger et al. [88] using a fluorescence detector (Figure A1).

### 4.6. Pyrrolizidine Alkaloid Extraction and Purification

Samples (100 mg) of freeze-dried HRs were pulverized in a mortar and pestle and transferred together with 10 mL 0.05 M H_2_SO_4_ containing 20 µg of the PA heliotrine as an internal standard into 15-mL tubes to be extracted overnight under constant end-over-end agitation (30 rpm) at room temperature. The supernatant obtained by centrifugation at 10,000× *g* for 15 min was incubated with 100 mg zinc dust for an additional four hours to reduce the PA *N-*oxides for GC-MS analyses according to Kempf et al. [89]. Once the added zinc had been removed by centrifugation, the supernatant was purified by solid phase extraction (Strata-SCX© cartridges, Phenomenex, Torrance, CA, USA) as described previously [37]. After elution with 1 mL ammoniac methanol (5%, *v*/*v*), the sample was dried under an air stream and reconstituted in 100 μL methanol for GC-MS analysis.

### 4.7. GC-MS Analysis

**Figure A2:**
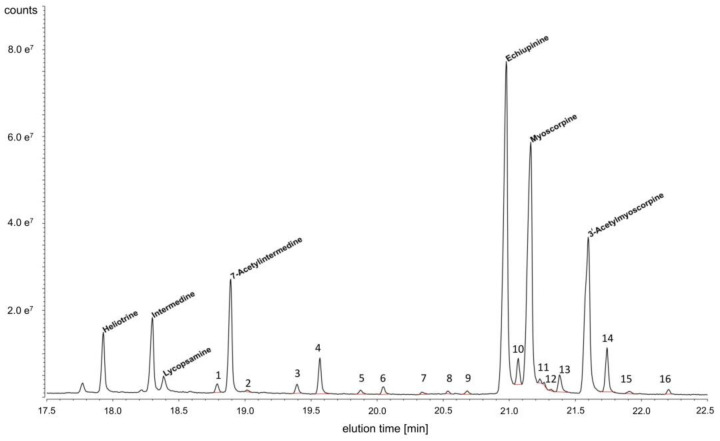
GC-MS total ion chromatogram of a purified extract of hairy root line HR-CT1. Major peaks were identified as ehiupinine (~32% of total PA content), myoscorpine (~27%), 3’-acetylmyoscorpine (~18%), 7-acetylintermedine (~10%), and intermedine (~7%). All numbered peaks indicate unidentified PAs with low abundance (~5%).

GC-MS measurements were carried out in a Trace1300/1310 GC system coupled to a Triple Quadrupole Mass Spectrometer (TSQ Duo, Thermo Fisher Scientific) equipped with a split/splitless (SSL) injector. The applied GC conditions were as follows: injector temperature: 280 °C; temperature program: 100 °C for 3 min, 100 °C to 300 °C at 20 °C/min, final hold at 300 °C for 10 min; carrier gas: helium with a flow of 1 mL/min; injection mode: split ratio 10; MS transfer line: 300 °C. EI-MS-spectra were recorded at 70 eV and an ion source temperature of 280 °C. PA identification was based on comparison with standard compounds analyzed under the same experimental conditions, and Kovats indices (R_i_ values), spectra, molecular ions, and MS fragmentation patterns were compared with the NIST database, the MS data base of PAs of our institute, and the literature. For the quantification of PAs in the HR lines, we considered the five most abundant alkaloids, i.e., intermedine, 7-actylintermedine, echiupinine, myoscorpine, and 3′-acetylmyoscorpine. Further PAs were detectable but did not contribute significantly to the total amount of PAs (Figure A2).

### 4.8. Feeding of Homospermidine to HR Lines with Knocked-Out hss Gene

Homospermidine was prepared from putrescine enzymatically by using bacterial HSS and purified by ion exchange chromatography [90]. The resulting homospermidine was applied to the HR-A14 with the knocked-out *hss* gene at a concentration of 1 mM in 10 mL liquid MS20 medium (supplemented with a mixture of ticarcillin and clavulanic acid, see above) containing approx. 0.5 g fresh weight of this HR line. Preparations were grown in the dark at 21 °C on an orbital shaker at 130 rpm. After 14 days, the complete HR culture was transferred to 70 mL fresh MS20 media without homospermidine, and growth was continued for another 14 days. A total culturing time of four weeks was used to guarantee maximum turnover, because previous studies had indicated a slow accumulation of PA-intermediates in the HRs of *S. officinale* [79]. At the end of the experiment, the HRs were harvested, washed with water, patted dry with tissue paper, and freeze-dried for PA extraction as described above.

### 4.9. Statistical Analyses

Statistical analyses were performed in GraphPad Prism 8. Differences in homospermidine and PA content were first tested for normal distribution by applying the Shapiro–Wilk test. The Tukey’s multiple comparisons test was used to determine the relative differences in homospermidine and PA content, respectively, for KD and KO lines in comparison with control lines. Also, parametric unpaired *t*-test was used to compare each group to the control group separately.

## Figures and Tables

**Table 1:** Sequence analyses of CRISPR/Cas9-induced mutations by construct A in exon 3 and by construct B in exon 7 of the *hss* gene in transgenic HRs of comfrey. For all analyzed lines, the 20 nt-long protospacer sequences with the attached PAM motif (TGG, bold) are shown in comparison with the sequence of the *hss* gene of the control line (HR-CT1) resulting from transformation with the empty vector. The sequence for the *hss* gene of HR-CT1 is identical to the unmodified *hss* gene of the wild-type (WT). The recognition sequence motives for the endonucleases *Pvu*II (A, 5′-CAGCTG-3′) and *Bsa*JI (B, 5′-CCNNGG-3′) are labeled in red. The red arrow head indicates the position, to which Cas9 was directed by the sgRNA in order to introduce the double-strand break. Dashes indicate deletions, blue nucleotides insertions. Predicted effects on the *hss* gene: KO, knock out, KD, knock down, NE, no editing, n.a., not analyzed. The screen for the presence of PAs refers to only one technical replicate, while established effects are given for lines of which three technical replicates have been analyzed (Figure 3a).

Construct	Sequence of Protospacer Motif Including PAM	Size of Del./Ins.	Allelic Differences of Mutations	Expected Effect on *hss*	PA Screen	Established Effect
**A (exon 3)**						
HR-CT1	GTGATGGTTACAA CAGC_▲_TGG **TGG**	0	WT	NE	+	NE
HR-A1	GTGATGGTTACAA ----_▲_TGG **TGG**	−4	Homozygous	KO	−	n.a.
HR-A3	GTGATGGTTACAA CAGC_▲_TGG **TGG**	0	WT	NE	+	n.a.
HR-A4	GTGATGGTTACAA ----_▲_TGG **TGG**	−4	Homozygous	KO	−	KO
HR-A5	------------- ----_▲_— --**---** GTGATGG------ ----_▲_— --**---**	−52 −78	Biallelicprotospacer completely lost because of a large deletion in one of the alleles	KO	−	KO
HR-A6	GTGATGGTTACAA CA--_▲_TGG **TGG**	−2	Homozygous	KO	−	KO
HR-A14	GTGATGGTT ---- ----_▲_-GG **TGG**	−9	Homozygous	NE, KD, KO	−	KO
HR-A16	GTG---------- ----_▲_--G **TGG**	−16	Homozygous	KO	−	KO
HR-A17	GTGATGGTTACAA C---_▲_TGG **TGG**	−3	Monoallelic	NE, KD	+	n.a.
HR-A18	GTGATGGTTACAA C---_▲_TGG **TGG**	−3	Monoallelic	NE, KD	+	n.a.
**B (exon 7)**						
HR-CT1	ACAGGGATAATAAT CCT_▲_GGG **TGG**	0	WT	NE	+	NE
HR-B2	ACAGGGATAA ---- ---_▲_GGG **TGG**	−7	Monoallelic	KD	+	KD
HR-B4	ACAGGGATAATAAT CCT T _▲_GGG **TGG**	+1	Monoallelic	KD	+	n.a.
HR-B5	ACAGGGATAATAAT -CT_▲_GGG **TGG**	−1	Monoallelic	KD	+	KD
HR-B6	ACAGGGATA ----- ---_▲_GGG **TGG**	−8	Monoallelic	KD	+	KD
HR-B7	ACAGGGATAATAAT CCT T _▲_GGG **TGG**	+1	Monoallelic	KD	+	KD
HR-B8	ACAGGGA ------- --T_▲_GGG **TGG**	−9	Monoallelic	NE, KD	+	n.a.
HR-B9	ACAGGGATAATAAT CCT T _▲_GGG **TGG**	+1	Monoallelic	KD	+	KD

## Data Availability

The sequence reported in this paper has been submitted to GenBank with the accession no. MW365954 for the *S. officinale* genomic sequence of homospermidine synthase.

## Supplementary Materials

The following are available online, Figure S1: Test for the identity of analyzed root lines to be HRs. Figure S2: Images of selected HR lines of S. officinale.

## Appendix A

**Table A1:** Primer sequences

Nr.	5‘-sequence-3‘	Purpose
P1	ATATAAGCTTATCATACATGAGAATTAAGGGAGTCAC	Amplification of the hygromycin resistance gene, HindIII restriction site underlined
P2	ATATAAGCTTATCAGCTTGCATGCCGGTCGATCTAG	Amplification of the hygromycin resistance gene, HindIII restriction site underlined
P3	TACTCGAGATGGGGGAAGTAGCCGCTGCT	Amplification of the complete *hss* gene, Start ATG underlined.
P4	TATGATCATCACTTAGATAAATTATTTGCCTGTTT	Amplification of the complete *hss* gene, Stop codon underlined.
P5	ATTGGTGATGGTTACAACAGCTGG	N_20_ oligos for motif in exon 3, overhang for ligation underlined
P6	AAACCCAGCTGTTGTAACCATCAC	N_20_ oligos for motif in exon 3, overhang for ligation underlined
P7	ATTGACAGGGATAATAATCCTGGG	N_20_ oligos for motif in exon 7, overhang for ligation underlined
P8	AAACCCCAGGATTATTATCCCTGT	N_20_ oligos for motif in exon 7, overhang for ligation underlined
P9	ATTGATGGAAGTGATTCTGGCGCC	N_20_ oligos for motif in exon 8, overhang for ligation underlined
P10	AAACGGCGCCAGAATCACTTCCAT	N_20_ oligos for motif in exon 8, overhang for ligation underlined
P11	GTCTTTCACCTCTCTTTGGTTA	Sequencing of destination vector constructs
P12	AAGCTTGCATGCCTGCAGGT	Sequencing of destination vector constructs
P13	TTCCGGACTGCGTGAGACAT	Amplification of genomic region exon 2 to 4 to test for mutations
P14	CATCTTGTCCAAAATGTT	Amplification of genomic region exon 2 to 4 to test for mutations
P15	AGTGCTATGGACAATGAATCAGTGA	Amplification of genomic region exon 7 to 8 to test for mutations
P16	CCTAACTTTCTTGGCGTTTTCG	Amplification of genomic region exon 7 to 8 to test for mutations
P17	ACGGTGAGTGTGGTTGTAGG	Amplification of rolA gene acc. to [43]
P18	GCCACGTGCGTATTAATCCC	Amplification of rolA gene acc. to [43]
P19	ATGTCGCAAGGACGTAAGCCCA	Amplification of virD gene acc. to [43]
P20	GGAGTCTTTCAGCATGGAGCAA	Amplification of virD gene acc. to [43]

## References

[1] Jank B., Rath J. (2017). The risk of pyrrolizidine alkaloids in human food and animal feed. Trends Plant. Sci..

[2] Schrenk D., Gao L., Lin G., Mahony C., Mulder P.P.J., Peijnenburg A., Pfuhler S., Rietjens I.M.C.M., Rutz L., Steinhoff B. (2020). Pyrrolizidine alkaloids in food and phytomedicine: Occurrence, exposure, toxicity, mechanisms, and risk assessment—A review. Food Chem. Toxicol..

[3] Roeder E., Wiedenfeld H., Edgar J.A. (2015). Pyrrolizidine alkaloids in medicinal plants from North America. Die Pharm..

[4] Wiedenfeld H., Edgar J. (2011). Toxicity of pyrrolizidine alkaloids to humans and ruminants. Phytochem. Rev..

[5] Allgaier C., Franz S. (2015). Risk assessment on the use of herbal medicinal products containing pyrrolizidine alkaloids. Regul. Toxicol. Pharm..

[6] Lamberecht K. (2016). Pyrrolizidine alkaloids—Impact of the public statements made by EMA and national health authorities on the pharmaceutical industry. Master Thesis.

[7] Knutsen H.K., Alexander J., Barregard L., Bignami M., Brüschweiler B., Ceccatelli S., Cottrill B., Dinovi M., Edler L., Grasl-Kraupp B. (2017). Risks for human health related to the presence of pyrrolizidine alkaloids in honey, tea, herbal infusions and food supplements. EFSA J..

[8] Debrunner B., Meier B. (1998). Petasites hybridus: A tool for interdisciplinary research in phytotherapy. Pharm. Acta Helv..

[9] Kopp T., Abdel-Tawab M., Mizaikoff B. (2020). Extracting and analyzing pyrrolizidine alkaloids in medicinal plants: A review. Toxins.

[10] Staiger C. (2012). Comfrey: A clinical overview. Phytother. Res..

[11] Predel H.G., Giannetti B., Koll R., Bulitta M., Staiger C. (2005). Efficacy of a comfrey root extract ointment in comparison to a diclofenac gel in the treatment of ankle distortions: Results of an observer-blind, randomized, multicenter study. Phytomedicine.

[12] Seigner J., Junker-Samek M., Plaza A., D’Urso G., Masullo M., Piacente S., Holper-Schichl Y.M., de Martin R. (2019). A *Symphytum officinale* root extract exerts anti-inflammatory properties by affecting two distinct steps of NF-κB signaling. Front. Pharm..

[13] Avila C., Breakspear I., Hawrelak J., Salmond S., Evans S. (2020). A systematic review and quality assessment of case reports of adverse events for borage (*Borago officinalis*), coltsfoot (*Tussilago farfara*) and comfrey (*Symphytum officinale*). Fitoterapia.

[14] Fu P.P., Xia Q., Lin G., Chou M.W. (2004). Pyrrolizidine alkaloids—genotoxicity, metabolism enzymes, metabolic activation, and mechanisms. Drug Metab. Rev..

[15] Seremet O.C., Barbuceanu F., Ionica F.E., Margina D.M., GuTu C.M., Olaru O.T., Ilie M., Gonciar V., Negres S., ChiriTa C. (2016). Oral toxicity study of certain plant extracts containing pyrrolizidine alkaloids. Rom. J. Morphol. Embryol..

[16] Hartmann T., Ober D., Schaller A. (2008). Defense by pyrrolizidine alkaloids: Developed by plants and recruited by insects. Induced Plant Resistance to Herbivory.

[17] Hartmann T., Conner W.E. (2009). Pyrrolizidine alkaloids: The successful adoption of a plant chemical defense. Tiger Moths and Woolly Bears. Behavior, Ecology, and Evolution of the Arctiidae.

[18] Prakash A.S., Pereira T.N., Reilly P.E.B., Seawright A.A. (1999). Pyrrolizidine alkaloids in human diet. Mutat. Res..

[19] Kempf M., Heil S., Hasslauer I., Schmidt L., von der Ohe K., Theuring C., Reinhard A., Schreier P., Beuerle T. (2010). Pyrrolizidine alkaloids in pollen and pollen products. Mol. Nutr. Food Res..

[20] EFSA (2007). European Food Safety Authority Opinion of the scientific panel of contaminants in the food chain on a request from the european commission realted to pyrrolizidine alkaloids as undesirable substances in animal feed. Efsa J..

[21] Hartmann T., Witte L., Pelletier S.W. (1995). Chemistry, biology and chemoecology of the pyrrolizidine alkaloids. Alkaloids: Chemical and Biological Perspectives.

[22] Langel D., Ober D., Pelser P. (2011). The evolution of pyrrolizidine alkaloid biosynthesis and diversity in the Senecioneae. Phytochem. Rev..

[23] Chen T., Mei N., Fu P.P. (2010). Genotoxicity of pyrrolizidine alkaloids. J. Appl. Toxicol..

[24] Fu P.P., Xia Q., Lin G., Chou M.W. (2002). Genotoxic pyrrolizidine alkaloids—Mechanisms leading to DNA adduct formation and tumorigenicity. Int. J. Mol. Sci..

[25] Ober D., Hartmann T. (1999). Homospermidine synthase, the first pathway-specific enzyme of pyrrolizidine alkaloid biosynthesis, evolved from deoxyhypusine synthase. Proc. Natl. Acad. Sci. USA.

[26] Ober D., Kaltenegger E. (2009). Pyrrolizidine alkaloid biosynthesis, evolution of a pathway in plant secondary metabolism. Phytochemistry.

[27] Reimann A., Nurhayati N., Backenköhler A., Ober D. (2004). Repeated evolution of the pyrrolizidine alkaloid-mediated defense system in separate angiosperm lineages. Plant. Cell.

[28] Kaltenegger E., Eich E., Ober D. (2013). Evolution of homospermidine synthase in the Convolvulaceae: A story of gene duplication, gene loss, and periods of various selection pressures. Plant. Cell.

[29] Irmer S., Podzun N., Langel D., Heidemann F., Kaltenegger E., Schemmerling B., Geilfus C.-M., Zorb C., Ober D. (2015). New aspect of plant-rhizobia interaction: Alkaloid biosynthesis in *Crotalaria* depends on nodulation. Proc. Natl. Acad. Sci. USA.

[30] Ober D., Hartmann T. (1999). Deoxyhypusine synthase from tobacco. cDNA isolation, characterization, and bacterial expression of an enzyme with extended substrate specificity. J. Biol. Chem..

[31] Ober D., Hartmann T. (2000). Phylogenetic origin of a secondary pathway: The case of pyrrolizidine alkaloids. Plant. Mol. Biol..

[32] Böttcher F., Adolph R.D., Hartmann T. (1993). Homospermidine synthase, the first pathway-specific enzyme in pyrrolizidine alkaloid biosynthesis. Phytochemistry.

[33] Moll S., Anke S., Kahmann U., Hänsch R., Hartmann T., Ober D. (2002). Cell-specific expression of homospermidine synthase, the entry enzyme of the pyrrolizidine alkaloid pathway in *Senecio vernalis*, in comparison with its ancestor, deoxyhypusine synthase. Plant Physiol..

[34] Anke S., Niemüller D., Moll S., Hänsch R., Ober D. (2004). Polyphyletic origin of pyrrolizidine alkaloids within the Asteraceae. Evidence from differential tissue expression of homospermidine synthase. Plant Physiol..

[35] Anke S., Gonde D., Kaltenegger E., Hansch R., Theuring C., Ober D. (2008). Pyrrolizidine alkaloid biosynthesis in *Phalaenopsis* orchids: Developmental expression of alkaloid-specific homospermidine synthase in root tips and young flower buds. Plant Physiol..

[36] Niemüller D., Reimann A., Ober D. (2012). Distinct cell-specific expression of homospermidine synthase involved in pyrrolizidine alkaloid biosynthesis in three species of the Boraginales. Plant Physiol..

[37] Kruse L.H., Stegemann T., Sievert C., Ober D. (2017). Identification of a second site of pyrrolizidine alkaloid biosynthesis in Comfrey to boost plant defense in floral stage. Plant Physiol..

[38] DeBoer K.D., Dalton H.L., Edward F.J., Hamill J.D. (2011). RNAi-mediated downregulation of ornithine decarboxylase (ODC) leads to reduced nicotine and increased anatabine levels in transgenic *Nicotiana tabacum* L. Phytochemistry.

[39] Glenn W.S., Runguphan W., O’Connor S.E. (2013). Recent progress in the metabolic engineering of alkaloids in plant systems. Curr. Opin. Biotechnol..

[40] Runguphan W., Maresh J.J., O’Connor S.E. (2009). Silencing of tryptamine biosynthesis for production of nonnatural alkaloids in plant culture. Proc. Natl. Acad. Sci. USA.

[41] Yuan L., Grotewold E. (2015). Metabolic engineering to enhance the value of plants as green factories. Metab. Eng..

[42] Pandey P., Senthil-Kumar M., Mysore K.S., Senthil-Kumar M., Mysore K.S. (2015). Advances in Plant Gene Silencing Methods. Plant Gene Silencing.

[43] Kruse L.H., Stegemann T., Jensen-Kroll J., Engelhardt A., Wesseling A.M., Lippert A., Ludwig-Müller J., Ober D. (2019). Reduction of pyrrolizidine alkaloid levels in comfrey (Symphytum officinale) hairy roots by RNAi silencing of homospermidine synthase. Planta Med..

[44] Agrawal N., Dasaradhi P.V., Mohmmed A., Malhotra P., Bhatnagar R.K., Mukherjee S.K. (2003). RNA interference: Biology, mechanism, and applications. Microbiol. Mol. Biol. Rev..

[45] Lu R., Martin-Hernandez A.M., Peart J.R., Malcuit I., Baulcombe D.C. (2003). Virus-induced gene silencing in plants. Methods.

[46] Osakabe Y., Osakabe K. (2015). Genome editing with engineered nucleases in plants. Plant Cell Physiol..

[47] Razzaq A., Saleem F., Kanwal M., Mustafa G., Yousaf S., Imran Arshad H.M., Hameed M.K., Khan M.S., Joyia F.A. (2019). Modern trends in plant genome editing: An inclusive review of the CRISPR/Cas9 toolbox. Int. J. Mol. Sci..

[48] Travis J. (2015). Making the cut. Science.

[49] Ledford H., Callaway E. (2020). Pioneers of CRISPR gene editing win chemistry Nobel Prize. Nature.

[50] Bortesi L., Fischer R. (2015). The CRISPR/Cas9 system for plant genome editing and beyond. Biotech. Adv..

[51] Puchta H. (2016). Using CRISPR/Cas in three dimensions: Towards synthetic plant genomes, transcriptomes and epigenomes. Plant J. Cell Mol. Biol..

[52] Puchta H. (2017). Applying CRISPR/Cas for genome engineering in plants: The best is yet to come. Curr. Opin. Plant Biol..

[53] Schiml S., Puchta H. (2016). Revolutionizing plant biology: Multiple ways of genome engineering by CRISPR/Cas. Plant Methods.

[54] Bewg W.P., Ci D., Tsai C.-J. (2018). Genome editing in trees: From multiple repair pathways to long-term stability. Front. Plant Sci..

[55] Schiml S., Fauser F., Puchta H. (2016). CRISPR/Cas-Mediated Site-Specific Mutagenesis in Arabidopsis thaliana Using Cas9 Nucleases and Paired Nickases. Methods Mol. Biol..

[56] Stegemann T., Kruse L.H., Brütt M., Ober D. (2018). Specific distribution of pyrrolizidine alkaloids in floral parts of comfrey (*Symphytum officinale*) and its implications for flower ecology. J. Chem. Ecol..

[57] Hartmann T. (2008). The lost origin of chemical ecology in the late 19th century. Proc. Natl. Acad. Sci. USA.

[58] Roeder E. (1995). Medicinal plants in Europe containing pyrrolizidine alkaloids. Pharmazie.

[59] Ikram N., Simonsen H.T. (2017). A review of biotechnological artemisinin production in plants. Front. Plant Sci..

[60] Wurtzel E.T., Vickers C.E., Hanson A.D., Millar A.H., Cooper M., Voss-Fels K.P., Nikel P.I., Erb T.J. (2019). Revolutionizing agriculture with synthetic biology. Nat. Plants.

[61] Rischer H., Szilvay G.R., Oksman-Caldentey K.M. (2020). Cellular agriculture—industrial biotechnology for food and materials. Curr. Opin. Biotechnol..

[62] Sun H., Liu Z., Zhao H., Ang E.L. (2015). Recent advances in combinatorial biosynthesis for drug discovery. Drug Des. Devel.

[63] Cravens A., Payne J., Smolke C.D. (2019). Synthetic biology strategies for microbial biosynthesis of plant natural products. Nat. Commun..

[64] McGinnis K.M. (2010). RNAi for functional genomics in plants. Brief. Funct. Genom..

[65] Gutierrez-Valdes N., Hakkinen S.T., Lemasson C., Guillet M., Oksman-Caldentey K.M., Ritala A., Cardon F. (2020). Hairy root cultures—A versatile tool with multiple applications. Front. Plant Sci..

[66] Doench J.G., Hartenian E., Graham D.B., Tothova Z., Hegde M., Smith I., Sullender M., Ebert B.L., Xavier R.J., Root D.E. (2014). Rational design of highly active sgRNAs for CRISPR-Cas9-mediated gene inactivation. Nat. Biotechnol..

[67] Ober D., Gibas L., Witte L., Hartmann T. (2003). Evidence for general occurrence of homospermidine in plants and its supposed origin as by-product of deoxyhypusine synthase. Phytochemistry.

[68] Abdelhady M.I.S., Beuerle T., Ober D. (2009). Homospermidine in transgenic tobacco results in considerably reduced spermidine levels but is not converted to pyrrolizidine alkaloid precursors. Plant Mol. Biol..

[69] Beaudoin G.A., Facchini P.J. (2014). Benzylisoquinoline alkaloid biosynthesis in opium poppy. Planta.

[70] Payne R.M., Xu D., Foureau E., Teto Carqueijeiro M.I., Oudin A., Bernonville T.D., Novak V., Burow M., Olsen C.E., Jones D.M. (2017). An NPF transporter exports a central monoterpene indole alkaloid intermediate from the vacuole. Nat. Plants.

[71] Dewey R.E., Xie J. (2013). Molecular genetics of alkaloid biosynthesis in *Nicotiana tabacum*. Phytochemistry.

[72] Dobritzsch M., Lübken T., Eschen-Lippold L., Gorzolka K., Blum E., Matern A., Marillonnet S., Böttcher C., Dräger B., Rosahl S. (2016). MATE transporter-dependent export of hydroxycinnamic acid amides. Plant Cell.

[73] Adebesin F., Widhalm J.R., Boachon B., Lefevre F., Pierman B., Lynch J.H., Alam I., Junqueira B., Benke R., Ray S. (2017). Emission of volatile organic compounds from petunia flowers is facilitated by an ABC transporter. Science.

[74] Grotewold E. (2008). Transcription factors for predictive plant metabolic engineering: Are we there yet?. Curr. Opin. Biotechnol..

[75] Zhai R., Wang Z., Zhang S., Meng G., Song L., Wang Z., Li P., Ma F., Xu L. (2015). Two MYB transcription factors regulate flavonoid biosynthesis in pear fruit (*Pyrus bretschneideri* Rehd.). J. Exp. Bot..

[76] Yamada Y., Sato F. (2013). Transcription factors in alkaloid biosynthesis. Int. Rev. Cell Mol. Biol..

[77] Srivastava S., Srivastava A.K. (2007). Hairy root culture for mass-production of high-value secondary metabolites. Crit. Rev. Biotechnol..

[78] Frölich C., Hartmann T., Ober D. (2006). Tissue distribution and biosynthesis of 1,2-saturated pyrrolizidine alkaloids in *Phalaenopsis* hybrides (Orchidaceae). Phytochemistry.

[79] Frölich C., Ober D., Hartmann T. (2007). Tissue distribution, core biosynthesis and diversification of pyrrolizidine alkaloids of the lycopsamine type in three Boraginaceae species. Phytochemistry.

[80] Sievert C., Beuerle T., Hollmann J., Ober D. (2015). Single cell subtractive transcriptomics for identification of cell-specifically expressed candidate genes of pyrrolizidine alkaloid biosynthesis. Phytochemistry.

[81] Li X., Jiang D.-H., Yong K., Zhang D.-B. (2007). Varied transcriptional efficiencies of multiple *Arabidopsis* U6 small nuclear RNA genes. J. Integr. Plant Biol..

[82] Waibel F., Filipowicz W. (1990). RNA-polymerase specificity of transcription of *Arabidopsis* U snRNA genes determined by promoter element spacing. Nature.

[83] Karimi M., Depicker A., Hilson P. (2007). Recombinational cloning with plant gateway vectors. Plant Physiol..

[84] Kuzma J., Nemecek-Marshall M., Pollock W.H., Fall R. (1995). Bacteria produce the volatile hydrocarbon isoprene. Curr. Microbiol.

[85] Wise A.A., Liu Z., Binns A.N. (2006). Three methods for the introduction of foreign DNA into *Agrobacterium*. Methods Mol. Biol..

[86] Murashige T., Skoog F. (1962). A revised medium for rapid growth and bio assays with tobacco tissue cultures. Physiol. Plant..

[87] Minocha R., Shortle W.C., Long S.L., Minocha S.C. (1994). A rapid and reliable procedure for extraction of cellular polyamines and inorganic ions from plant tissues. J. Plant Growth Regul..

[88] Kaltenegger E., Prakashrao A.S., Çiçek S.S., Ober D. (2021). Development of an activity assay for characterizing deoxyhypusine synthase and its diverse reaction products. Febs Open Bio.

[89] Kempf M., Beuerle T., Buhringer M., Denner M., Trost D., von der Ohe K., Bhavanam V.B., Schreier P. (2008). Pyrrolizidine alkaloids in honey: Risk analysis by gas chromatography-mass spectrometry. Mol. Nutr. Food Res..

[90] Graser G., Witte L., Robins D.J., Hartmann T. (1998). Incorporation of chirally deuterated putrescines into pyrrolizidine alkaloids: A reinvestigation. Phytochemistry.

