# Supplementary material for: CRISPR/Cas9-Mediated Genome Editing in Comfrey (Symphytum officinale) Hairy Roots Results in the Complete Eradication of Pyrrolizidine Alkaloids
Source: Molecules. 2021 Mar 10;26(6):1498. doi: 10.3390/molecules26061498 (PMC7998174; doi:10.3390/molecules26061498)
Supplement: Supplementary file 1 [file molecules-26-01498-s001.zip › 210226 Suppl Fig 1mod.pdf]

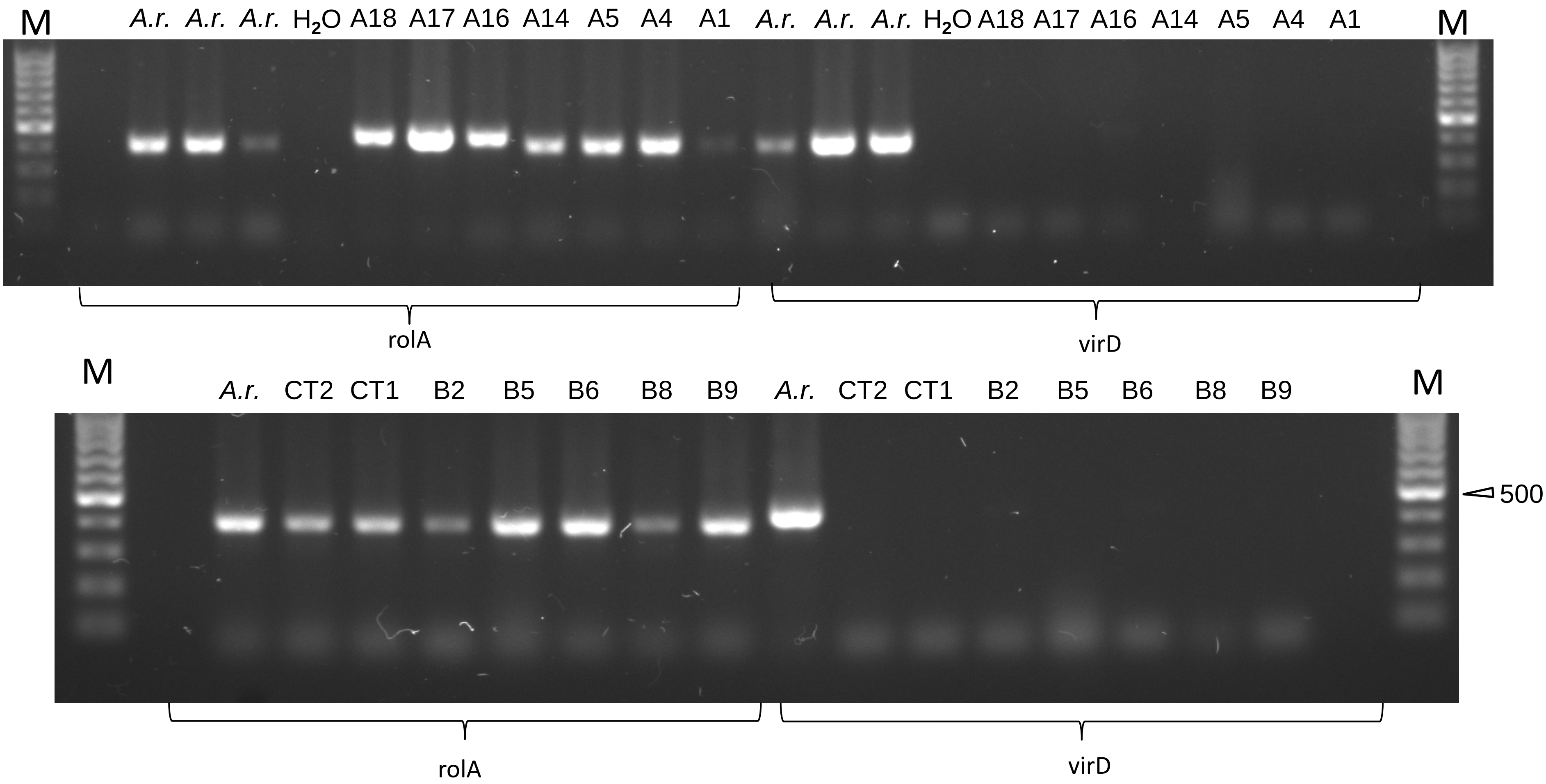

Figure S1: Test for the identity of analyzed lines to be HRs. PCR amplification of *rolA* and *virD* genes from genomic DNA isolated from HR lines that survived the hygromycin selection after separation on a 1.5% agarose gel. Successful integration of the *rolA* gene in the absence of the *virD* gene that is not transferred into the plant's genome confirmed the identity of the root lines as HRs and the absence of persisting agrobacteria. Given are the names of the HR lines used in this study. *A.r.*, DNA of *A. rhizogenes*, H_2O, no template control, M, GeneRuler 100 bp DNA ladder.
