# Supplementary material for: CRISPR/Cas9-Mediated Genome Editing in Comfrey (Symphytum officinale) Hairy Roots Results in the Complete Eradication of Pyrrolizidine Alkaloids
Source: Molecules. 2021 Mar 10;26(6):1498. doi: 10.3390/molecules26061498 (PMC7998174; doi:10.3390/molecules26061498)
Supplement: Supplementary file 1 [file molecules-26-01498-s001.zip › 210301 Suppl Fig 2.pdf]

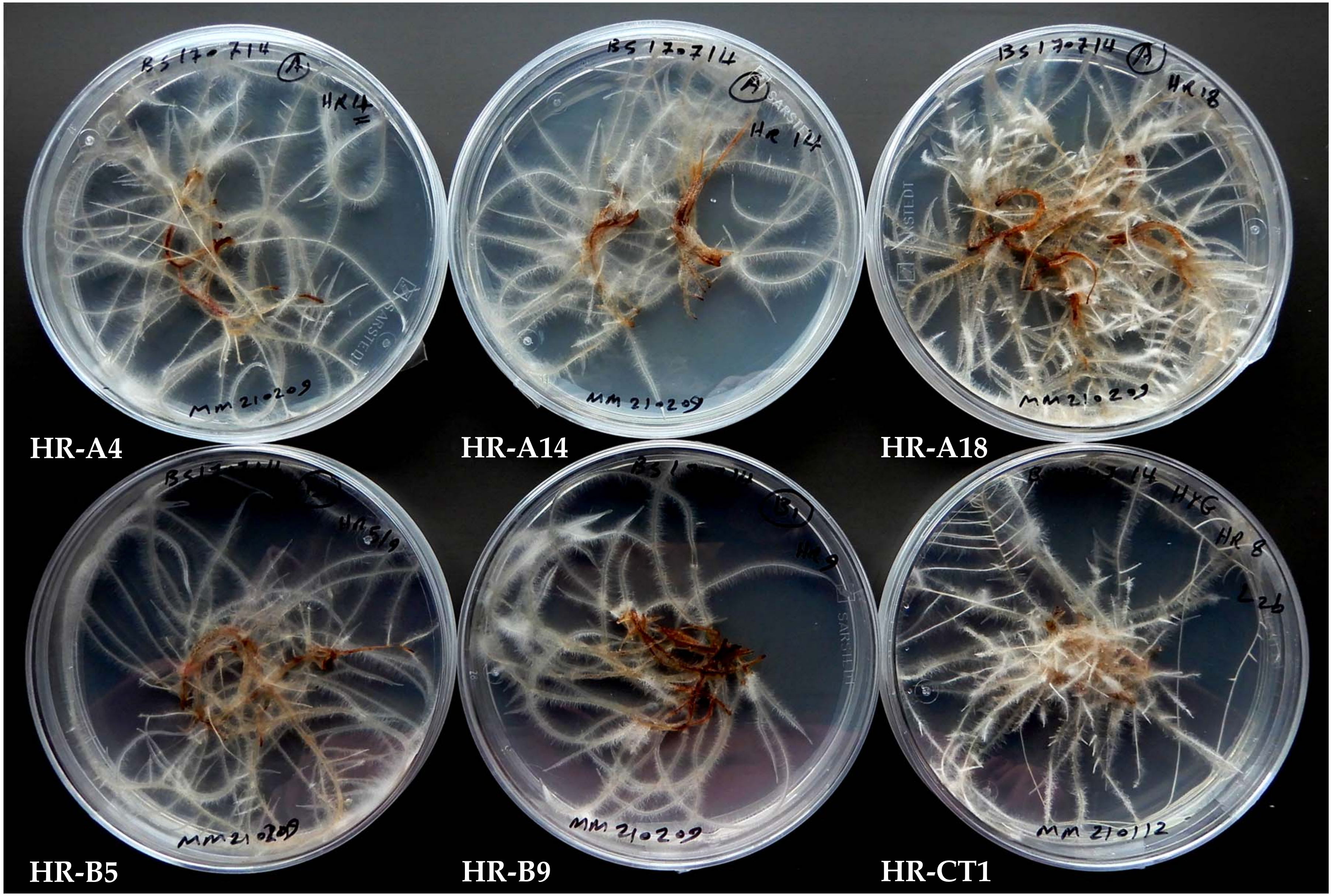

Figure S2: Images of selected HR lines of *S. officinale*. The HRs resulted from transformation of *S. officinale* leaves with *A. rhizogenes* carrying construct A (HR-A4, HR-A14, HR-A18), construct B (HR-B5, HR-B9), or the empty vector (HR-CT1). After selection of hygromycin-containing medium they were cultivated on a modified MS medium without hormones (MS20).
